# O’Reilly Prize

**Published:** 1868-10

**Authors:** 


### O'Reilly Prize.

Dr. John O'Reilly, of New York, having offered, through the N. Y. Academy of Medicine, a Prize of six hundred dollars for an Essay on the Physiology and Pathology of the Sympathetic or Ganglionic Nervous System, the Committee of Award, appointed by the Council of the Academy, have adopted, with the concurrence of the Council, the following Regulations:

I. The competing Essays shall be sent in to the Chairman of the Committee, Prof. J. C. Dalton, M. D., No. 101 East Twenty-third street, New York, on or before the first day of March, 1869.

II. Each Essay shall be marked with some distinguishing device or motto, and accompanied by a sealed envelope bearing the same device or motto, and containing the name and address of the writer.

III. The Essay selected by the Committee shall be transmitted by them, together with its accompanying envelope, to the Council of the N. Y. Academy of Medicine, under whose direction the envelope shall be opened, and the name of the writer announced at the first meeting of the Academy, in May, 1869.

IV. This Prize is open for universal Competition.

V. The Committee have a right to reject whatever does not come up to a proper standard of merit.

ALFRED C. POST, M. D., Pres't of the Academy.

On behalf of the Council.

COMMITTEE OF AWARDS.

J. C. Dalton, M. D., Professor of Physiology, in the College of Physicians and Surgeons, New York.

A. Flint, Jr., M. D., Professor of Physiology, in the Bellevue Hospital Medical College, New York.

Alfred L. Loomis, M. D., Professor of the Institutes and Practice of Medicine in the University Medical College, New York.

New York, December, 1867.

